# Effects of Remote Patient Monitoring on Health Care Utilization in Patients With Noncommunicable Diseases: Systematic Review and Meta-Analysis

**DOI:** 10.2196/68464

**Published:** 2025-10-01

**Authors:** Geir Smedslund, Nina Østerås, Christine Hillestad Hestevik

**Affiliations:** 1 Centre for Treatment of Rheumatic and Musculoskeletal Diseases (REMEDY) Diakonhjemmet Hospital Oslo Norway; 2 Health and Social Care Interventions Division of Health Services Norwegian Institute of Public Health Oslo Norway

**Keywords:** remote patient monitoring, health care resource utilization, noncommunicable diseases, systematic review, hospitalizations, outpatient visits, remote consultation, chronic disease, patient monitoring, length of stay, emergency service, hospital

## Abstract

**Background:**

Management of noncommunicable diseases (NCDs) is an increasing challenge for health care systems. Although remote patient monitoring presents a promising solution by utilizing technology to monitor patients outside clinical settings, there is a lack of knowledge about the effect on resource utilization.

**Objective:**

This systematic review aimed to review the effects of remote patient monitoring on health care resource utilization by patients with NCDs.

**Methods:**

Eligible randomized controlled trials (RCTs) involved digital transmission of health data from patients to health care personnel. Outcomes included hospitalizations, length of stay, outpatient visits, and emergency visits. A systematic literature search was performed in Medline, Embase, and Cochrane Central Register of Controlled Trials in June 2024. Titles, abstracts, and full texts were screened individually by 2 authors. Risk of bias was assessed, and data were extracted, analyzed, and pooled in meta-analysis when possible. Confidence in the estimates was assessed using the Grading of Recommendations Assessment, Development and Evaluation (GRADE) approach.

**Results:**

We included 40 RCTs published between 2017 and 2024. The largest group of NCDs was cardiovascular disease (16 studies). Remote patient monitoring may slightly decrease the proportion of hospitalizations compared with usual care (risk ratio [RR] 0.86, 95% CI 0.77 to 0.95; low certainty). Compared with usual care, remote patient monitoring had fewer or an equal number of hospitalizations (mean difference –0.13, 95% CI –0.29 to 0.03; low certainty). Hospital length of stay may be slightly reduced with remote patient monitoring compared with usual care (mean difference –0.84, 95% CI –1.61 to –0.06 days; low certainty). The proportion of outpatient visits showed probably little to no difference between remote patient monitoring and usual care (RR 0.94, 95% CI 0.87 to 1.02; moderate certainty). Compared with usual care, remote patient monitoring had slightly more outpatient visits, but the CI was wide (mean difference 0.41, 95% CI –0.22 to 1.03; low certainty). The results indicate a small or no difference between remote patient monitoring and usual care regarding proportion of emergency visits (RR 0.91, 95% CI 0.79 to 1.05; low certainty). We are uncertain whether remote patient monitoring increases or decreases the number of emergency visits, as the evidence was of very low certainty.

**Conclusions:**

This systematic review showed that remote patient monitoring possibly led to lower proportions of patients being hospitalized, fewer hospitalizations, and shorter hospital length of stay compared with usual care. Patients undergoing remote monitoring had possibly more outpatient visits compared with usual care. The proportions of patients with outpatient visits or emergency visits were probably similar. Finally, we had very low certainty in the number of emergency visits. The results should be considered with caution as the certainty of evidence was moderate to very low. We did not find results regarding institutional stay.

## Introduction

Noncommunicable diseases (NCDs) including diabetes, cardiovascular diseases, chronic respiratory diseases, and mental health conditions lead to widespread human suffering globally and impose a high burden on health care systems, both financially and structurally [1,2]. More than 70% of all deaths globally are caused by NCDs, and cardiovascular disease accounts for most of the deaths [3]. The already substantial societal costs of NCDs are expected to rise even further in the years to come [4]. NCDs contribute to reduced productivity, shortened working lives, and premature mortality, with estimates suggesting a cumulative global loss of between US $30 trillion and US $47 trillion in economic output between 2011 and 2030 [5]. Furthermore, NCDs lead to high treatment costs, imposing a direct economic burden on health systems [1,6].

The follow-up of these individuals is resource-intensive and requires a high degree of interdisciplinary and holistic care over time. This leads to increased demands on both capacity and competence in health care services, as these patients often need both continuous monitoring and acute interventions [1,2]. As a result, there is significant interest in exploring and testing new care models aimed at extending the services to patients’ homes through innovative technologies, which may contribute to preventing avoidable use of health care resources [7-9]. Welfare technology is highlighted as an important element in the development of the health and care sector [9].

Among these technologies, remote patient monitoring presents significant opportunities to improve the timeliness of care, enhance health outcomes, and potentially reduce unnecessary use of health care resources [9,10]. Remote patient monitoring utilizes technological tools to track patients’ health status outside of typical clinical settings, including patients’ homes or other remote locations. This method involves sending health data directly to health care providers via automated electronic systems or web- and phone-based interfaces, facilitating timely interventions if health status deteriorates [11,12].

This process ensures that health care providers can respond rapidly and potentially prevent hospitalizations. Unlike traditional calendar-based hospital follow-up appointments, remote monitoring enables customized treatment plans tailored to each patient's specific needs and disease activity [11,12]. Routine monitoring of patient’s health data and receiving alerts when measurements surpass a predetermined threshold can enable health care providers to intervene promptly. This approach offers potential advantages for both individuals and society by lessening the burden on patients and decreasing the need for unnecessary medical visits [10,13].

Moreover, the use of welfare technology hopefully can free up resources within health care services, allowing them to be allocated more effectively to patients with greater needs. However, findings from studies examining patients’ utilization of health care services with remote patient monitoring compared with usual care have shown mixed results [14]. Therefore, we lack definitive knowledge about the effect of remote monitoring on resource utilization. [14] The purpose of this systematic review was to examine the effects of remote patient monitoring on health care resource utilization for individuals with NCDs, compared with usual care. The protocol for this review was preregistered in PROSPERO (CRD42023431366) on June 19, 2023. This review is an update of a previous review [15] published in Norwegian with the key messages and executive summary provided in English.

## Methods

We conducted a systematic review according to the guidelines described in the Cochrane handbook [16] and the PRISMA (Preferred Reporting Items for Systematic Reviews and Meta-Analyses) guidelines [17]. The completed PRISMA checklist is provided in Multimedia Appendix 1.

### Selection Criteria

Eligible studies were randomized controlled trials investigating the effect of remote monitoring of patients with NCDs (including chronic pulmonary diseases, diabetes, cardiovascular disease, cancer, mental health disorders, and comorbidities) on health care utilization, compared with usual care, published in English or a Scandinavian language.

The interventions had to involve digital transmission of health data from the patient to health care personnel, who would then contact the patient and provide medical support and guidance if the measurements indicated signs of deterioration or fell outside normal values. We also included studies that incorporated additional components alongside remote patient monitoring, such as rehabilitation guidance, exercise prescriptions, and other forms of support.

Studies involving digital follow-up without any element of self-reporting, such as implants that transmit data automatically, were excluded.

Given the rapid advancements in digital health technologies, including remote monitoring tools, software platforms, and communication infrastructure, we prioritized newer studies to ensure the findings reflected current clinical practice and technological capabilities. Older studies may be based on outdated technologies or care models that no longer represent the current standard of care, potentially limiting the applicability and relevance of their results. Hence, only studies published from 2017 and onwards with interventions conducted in 2013 or later (within the last 10 years at the time of our first systematic search) were included.

The studies had to report one or more of the following outcomes: hospitalizations (proportions or numbers), length of hospital stay, outpatient consultations (proportions or numbers), use of emergency services (proportions or numbers), visits to general practitioners, use of home health care services, and use of institutional stay.

### Search Strategy

A librarian developed a search strategy corresponding to the inclusion criteria and searched the following databases: Medline (Ovid), Embase (Ovid) and Cochrane Central Register of Controlled Trials (Wiley). Searches were conducted in 3 rounds: April 2022, May 2023, and June 2024 (Multimedia Appendix 2).

### Inclusion Process

The authors independently screened the references identified in the previous review and the updated searches. We utilized the “priority screening” function in EPPI-Reviewer [18] to enhance the efficiency of screening titles and abstracts. This machine learning feature identifies the characteristics of included and excluded studies and predicts the likelihood of relevance for each reference. Priority screening prioritizes relevant records at the beginning of the screening process and relegates irrelevant records toward the end. Two authors (GS and CHH) independently screened the titles and abstracts to determine their eligibility according to our inclusion criteria. Disagreements were solved through discussion. We decided to stop screening after assessing 400 titles and abstracts in a row without including any additional references, as the inclusion rate had plateaued, suggesting that the algorithm had already identified all relevant studies

Subsequently, we obtained the full texts of references deemed relevant. In pairs, the authors (GS, CHH, NØ) then independently reviewed these full texts and made a final assessment to determine which studies to include. Disagreements were solved through consensus.

### Risk of Bias

Two authors (CHH and GS) independently assessed risk of bias for each outcome with the Cochrane risk-of-bias tool (RoB 2) for randomized trials [19]. Disagreements were solved through discussion. RoB 2 assesses risk of bias within 5 domains: randomization process, deviations from the intended interventions, missing outcome data, measurement of the outcome, and selection of the reported result. Overall risk of bias for each outcome was classified as low risk, some concerns, or high risk based on the domain with the highest risk of bias.

### Data Extraction

One author (GS) extracted data from the included studies, and another (CHH or NØ) checked the data against the publication. We extracted the following data: study and participant characteristics, information about the interventions, observation period, and outcomes related to resource use. These outcomes included hospitalizations (including readmissions), length of hospital stay, and number of outpatient visits (including clinic visits, outpatient visits, and medical consultations with private physicians and general practitioners), and emergency services. In case of missing information, we contacted the study authors.

### Statistical Analysis

We conducted meta-analyses combining studies reporting on the same outcome in RevMan Web [20]. Some of the effect sizes were transformed, as the original data were not reported in a format ready for effect size calculation. For dichotomous outcomes (proportion hospitalized, proportion of outpatient visits, proportion with emergency visits) we performed random-effects analyses with the risk ratio (RR) as the measure of relative effect. For continuous outcomes (number of hospitalizations, hospital length of stay, number of outpatient visits, number of emergency visits) we performed random-effects analyses with the mean difference as the measure of relative effect. A decision taken after the protocol was published was to add results of absolute effects on all outcomes to enhance interpretability and clinical relevance. We believe this can reduce the risk of misinterpreting large relative effects with low absolute benefit and facilitate meaningful comparisons and meta-analyses across studies with varying baseline risks

We did not plan any subgroup analyses or meta-regression in the protocol, but we did the following post hoc analyses: If there were 2 or more studies with the same NCD for an outcome, we subgrouped the meta-analysis per disease group. If there were 10 or more studies in a meta-analysis, we performed mixed effects meta-regression using the R-package [metafor] [21]. Explanatory variables were mean age and publication year. In addition, if there were 10 or more studies in a meta-analysis, we produced funnel plots and performed the Egger test in R [metafor].

### Grading of Recommendations Assessment, Development, and Evaluation

We used the Grading of Recommendations Assessment, Development, and Evaluation (GRADE) approach [22] to assess our certainty of the effect estimates per outcome within the following 5 domains: risk of bias, inconsistency, indirectness, imprecision, and publication bias. The GRADE approach specifies 4 levels of certainty: high, moderate, low, or very low. Table 1 illustrates how the certainty of the evidence and the importance of the outcome influence the description of the direction and size of an intervention’s effect.

**Table 1:** How certainty of the evidence and importance of the outcome influence the description of an intervention’s effect, as described in [23].

Level of certainty	Important benefit or harm	Less important benefit or harm	No important benefit or harm or null effect
High certainty	Increases or decreases	Increases or decreases slightly	Little to no difference
Moderate certainty	Probably increases or decreases	Probably increases or decreases slightly	Probably little to no difference
Low certainty	May increase or decrease	May increase or decrease slightly	May make little to no difference
Very low certainty	Uncertain whether intervention increases or decreases outcome	Uncertain whether intervention increases or decreases outcome	Uncertain whether intervention increases or decreases outcome

## Results

### Included Studies

**Figure 1:**
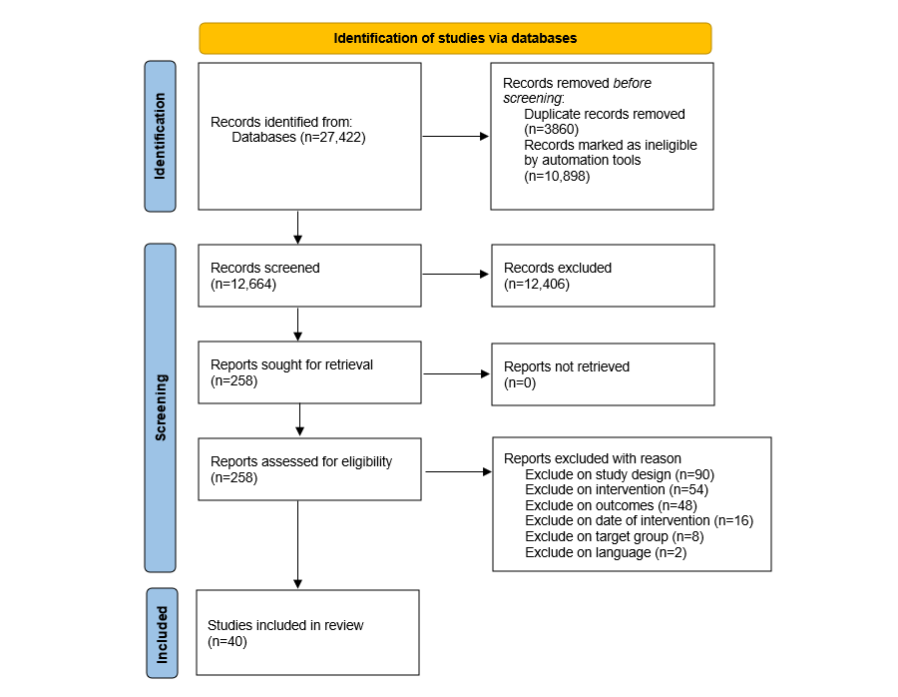
Flowchart of study selection process.

We identified 23,562 unique references in the literature search, of which 258 studies were evaluated in full text. In total, 218 of these studies were excluded for the following reasons: wrong study design (n=90), wrong intervention (n=54), wrong outcomes (n=48), observation period before 2013 (n=16), wrong target group (n=8), and wrong language (n=2; Figure 1).

We included 40 studies [24-62] published between 2017 and 2024 (Tables 2 and 3).

The studies were distributed across various countries, mainly representing high-income countries, with the highest number conducted in the United States and Denmark (Table 2).

The most frequently reported outcomes were proportion of hospitalized patients (including readmissions), proportion with emergency visits, and length of hospital stay.

**Table 2:** Key characteristics of included studies (N=40).

Characteristic		Details		
Total participants, n (range in individual studies)		11,617 (31-1225)		
Female participants (N=11,617), n (%)		4888 (42)		
Studies with mean age ≥50 years, n (%)		32 (80)		
**Chronic conditions** **,** **n** **(%) of studies**				
	Cardiovascular disease	16 (40)		
	Chronic obstructive pulmonary disease	8 (20)		
	Cancer	5 (13)		
	Multiple chronic conditions	4 (10)		
	Mental illness	3 (8)		
	Cystic fibrosis	2 (5)		
	Diabetes mellitus	2 (5)		
**Countries represented, n (%) of studies**				
	United States	5 (13)		
	Denmark	5 (13)		
	Canada	4 (10)		
	Spain	3 (8)		
	Australia	3 (8)		
	United Kingdom	2 (5)		
	Poland	2 (5)		
	Japan	2 (5)		
	Netherlands	1 (3)		
	Norway	1 (3)		
	Germany	1 (3)		
	Colombia	1 (3)		
	China	1 (3)		
	New Zealand	1 (3)		
	France	1 (3)		
	Russia	1 (3)		
	Singapore	1 (3)		
	Korea	1 (3)		
	Brazil	1 (3)		
	Argentina	1 (3)		
	Hong Kong	1 (3)		
	Multinational	1 (3)		

**Table 3:** Study characteristics (N=40).

First author (year)/country	Name of trial or program (app)	Sample size, n	Diagnoses	Age (years), mean (SD)	Women, %	Intervention	Relevant outcomes	Follow-up
Abelsen (2022)/Norway [63,64]	NR^a^	538	Chronic diseases (COPD^b^, T2DM^c^, HF^d^, cancer, mental illness)	69.7	49.0	Tablet for patients to answer simple questions about their health or to monitor measurements related to health status (such as BP^e^, blood glucose, oxygen saturation, or weight); automatic transfer of measurements from the home telemonitoring devices to the patient’s tablet and follow-up center	Number of hospitalizations, length of hospital stay, use of acute services, outpatient visits and home care services	6 and 12 months
Absolom (2021)/England [24]	App: eRAPID	508	Colorectal, breast, or gynecological cancers	56.0 (11.8)	79.9	An online eHealth system for patients to self‐report symptoms during cancer treatment; provision of automated severity-dependent patient advice guiding self-management or medical contact	Proportion hospitalized and proportion with emergency visits	6, 12, and 18 weeks
Achury-Saldana (2024)/Colombia [25]	App: ControlVit	175	HF	66 (11.9)	28.6	Daily (weight, arterial pressure, HR^f^, and symptoms) questionnaire; alerts when an increase in weight or change in HR or arterial pressure was detected	Proportion hospitalized	6 months
Ackermann (2021)/Australia [26]	Trial: MEL-SELF pilot study; app: ASICA^g^ Skin-Checker app	100	Localized melanoma	58.7 (12.0)	46	Usual care plus patient-led surveillance with instructional videos on how to perform SSE^h^, reminders to undertake SSE, a mobile dermatoscope attached to their smartphone, and an app that facilitated store-and-forward teledermatology and fast-tracked unscheduled clinic visits	Number of outpatient visits	6 months
Agarwal (2019)/Canada [27]	App: BlueStar	240	T2DM	51.8 (10.7)	48.0	Information related to T2DM management entered into the app by patients who received customized, evidence-based messages to impact motivation, behavior, and education; the transfer of health data to the user’s clinician also facilitated by the app	Proportion hospitalized and proportion with outpatient and emergency visits	3 and 6 months
Alshahrani (2024)/UK [28]	Trial: Tele-ACS study	337	Acute coronary syndrome	58.1	13.9	12-lead ECG^i^ belt with automatic monitoring of BP and pulse oximeter; remote consultation with clinician	Length of hospital stay and proportion with emergency visits	6 months
Andersen (2023)/Denmark [29]	NR	222	COPD	70 (64-76)^j^	61	Home measurement of oxygen saturation, HR, peak expiratory flow, body weight, and a questionnaire on dyspnea, cough, sputum volume, and color; results sent to a server with green, yellow, and red alerts	Proportion hospitalized, and length of hospital stay	6 and 24 months
Angermann (2023)/Germany [30]	Trial: randomized INH^k^ trial	715	Acute systolic HF	70 (60-78)^j^	29	Structured telephone monitoring based on patient-recorded vital signs and a 19-item standardized questionnaire addressing factors such as adverse events since previous contact, medication adherence, and satisfaction with remote patient monitoring	Proportion hospitalized	18 and 60 months
Benzo (2022)/US [31]	NR	375	COPD	69.0 (9.5)	56.5	Weekly HC^l^ calls and a remote monitoring system that had a computer tablet, an activity monitor, and an oximeter; tablet-gathered data transmitted to an online patient portal to monitor compliance with the rehabilitation routine and daily physical activity; aim of weekly HC call to be a behavior change intervention supported by the motivational interviewing and self-efficacy theories	Proportion hospitalized and with emergency visits	3 months
Broadbent (2018)/New Zealand [32]	App: iRobi robot	60	COPD	69.8 (10.1)	61.5	Robot provided to patients at home programmed to deliver COPD management consisting of several components guided by a clinical pathway; overall program designed to monitor health and prompt medical contact if health was deteriorating	Proportion hospitalized and hospital length of stay	4 months
Dorsch (2021)/US [33]	App: ManageHF4Life	83	Left ventricular ejection fraction	61.1 (9.0)	35.0	Self-monitoring with a mobile app, along with a Fitbit physical activity monitor and scale	Proportion hospitalized	12 weeks
Faurholt-Jepsen (2021)/Denmark [34]	Trial: RADMIS; app: Monsenso system smartphone app	98	Bipolar disorder	42.7 (13.5)	25.6	Daily self-monitoring of mood, level of activity, duration of sleep, and medication intake; collected smartphone data on a webpage regularly examined by study nurses who, based on the patients’ needs and collected data, contacted the patients and gave advice	Proportion hospitalized and duration of psychiatric readmissions	3 and 6 months
Franz (2022)/US [35]	Trial: eICE^m^ study	267	Cystic fibrosis	28.8% <18 years	50.9	Symptoms measured by patients using the CFRSD^n^ twice per week; study sites automatically alerted if a participant’s FEV1^o^ fell below baseline >10% or if CFRSD worsened in ≥2 of 8 respiratory symptoms	Proportion hospitalized, length of hospital stay, and number of outpatient and emergency visits per participant per year of follow-up	12 months
Galinier (2020)/France [36]	Trial: OSICAT	937	Acute HF	69.9 (12.4)	27.8	Telemonitoring program with 2 elements: electronic devices and a personalized educational component; telemonitoring group: identical set of electronic scales to measure body weight and a device to answer 8 symptom questions provided to each participant; measurement of body weight and recording of HF symptoms communicated daily to a secure server and analyzed automatically by an expert system that generated alerts, as necessary, with the aim of predicting episodes of cardiac decompensation	Proportion hospitalized and hospital length of stay	18 months
Garanin (2022)/Russia [37]	App: INME-01 tonometer	392	HF and acute coronary syndrome	66.3 (47.9)	49.4	Monitoring carried out with the certified INME-01 tonometer with an integrated GSM^p^ module that allows BP and pulse rate measurements and ability to transmit results via a cellular communication channel to a research center; based on the results of the received data, doctor able to contact the patient and adjust the previously prescribed treatment	Proportion hospitalized and length of hospital stay	3 months
Gomis-Pastor (2023)/Spain [38]	App: mHeart	134	Heart transplant	45 (16)	31	Multifaceted theory-based interventions provided during the study period to optimize therapy management using a mobile app	Proportion hospitalized and proportion with outpatient and emergency visits	6 and 12 months
Greer (2020)/US [39]	NR	181 (randomized)	Cancer	53.3 (12.9)	53.6	Smartphone mobile app with medication plan, reminders, symptom-reporting module, patient education, and Fitbit integration for tracking physical activity; used for 12 weeks	Number of hospitalizations and emergency visits	12 weeks
Hernandez-Quiles (2024)/Spain [40]	TELECARE	510	Advanced heart and lung disease	76 (14)	54.5	Monitoring of biological and medical questionnaires available in real time for the health care team; alarm routines	Proportion hospitalized, length of hospital stay, and proportion and number with emergency visits	45, 90, and 180 days
Indraratna (2022)/Australia [41]	App: TCC^q^ smartphone app	164	Acute coronary syndrome or HF	61.5 (12.5)	21.0	Smartphone app with Bluetooth-enabled devices for daily monitoring of weight, BP, and physical activity, along with educational push notifications	Proportion hospitalized	30 days
Jiang (2022)/Singapore [43]	App: NSSMP^r^	114	T2DM	52.8 (11.0)	30.0	6-month NSSMP consisting of an education session and a smartphone app for self-monitoring and recording of blood glucose, diet, and exercise	Number of hospitalizations and number of outpatient and emergency visits	6 months
Jiang (2024)/China [42]	NR	148	COPD with hypercapnic chronic respiratory failure	72.7 (6.79)	14.2	Internet of Things–based management based on telemonitoring of clinical and ventilator parameters for 12 months	Number with readmission	12 months
Krzesinski (2021)/Poland [44]	Trial: AMULET	605	HF and left ventricular ejection fraction	67 (14)^s^	21	Nurse-led noninvasive assessments supporting remote therapeutic decisions for patients after an episode of acute HF, including vital sign monitoring, assessments of HF symptoms, and physician recommendations	Proportion hospitalized	12 months
Laursen (2021)/Denmark [45]	Trial: ENTER; app: mDiary	78	Borderline personality disorder	26.8 (18.1)	87.0	Mobile diary app: psychoeducational material (dialectical behavior therapy) and visualizations of participant’s data; recording of symptom information (eg, emotional dysregulation and thoughts of suicide and self-harm) by participants so health care professionals could follow them in real time and use the registrations in the treatment	Proportion hospitalized and number of outpatient visits	1 month
Lee (2023)/Korea [46]	App: PRO‑CTCAE	222	Breast, lung, head and neck, esophageal, or gynecologic cancer	55.9 (9.7)	60.5	Completed symptom questionnaire every 7 days for 8 weeks; data accessible by clinicians using a web dashboard	Proportion hospitalized and proportion with outpatient and emergency visits (at least once)	2 months
Mizukawa (2019)/Japan [47]	NR	39	HF	72.4 (12.7)	48.7	3 arms: telemonitoring (collaborative management), self-management education, and usual care; all involved self-monitoring weight, BP, and pulse; 12-month disease management program also part of intervention groups; telemonitoring group: noninvasive physiologic telemonitoring devices to measure BP, pulse rate, and body weight for 12 months and data transmitted to the nurse’s computer; self-management group: described as “self-management”	Proportion hospitalized and length of hospital stay	24 months
Piotrowicz (2020)/Poland [48]	Trial: TELEREH-HF trial/CardioMessenger transmitter of the CareLink network, or erlin@home wireless transmitter	850	HF	62.4 (10.5)	11.4	Hybrid comprehensive telerehabilitation: supervised exercise training monitored with tele-ECG; monitoring of BP and body weight	Proportion hospitalized	9 weeks
Rohde (2024)/Brazil [49]	Trial: MESSAGE-HF study; app: Clever Care	699	HF (with reduced left ventricular ejection fraction [<40%])	61.2 (14.5)	34.2	Telemonitoring group: 4 daily short text messages to optimize self-care, active engagement, and early intervention; red flags based on feedback messages triggering automatic diuretic adjustment and/or telephone call from the health care team	Proportion hospitalized and with emergency visits	180 days
Shimoyama (2023)/Japan [50]	Trial: Telenursing system (COMPAS)	31	Chronic respiratory failure	73.0 (10.2)	40	Once a day, questions answered by each participant from a list of options; data on physical condition, including vital signs, respiratory symptoms, food intake, excretion, medication use, physical symptoms other than respiratory symptoms, and questions to the medical staff sent to the server and collected; transmitted data monitored remotely, and nursing support determined based on the results of each question item set in advanced consultation with the physician in charge	Number of hospitalizations and length of hospital stay	3 months
Soriano (2018)/Spain [51]	Trial: PROMETE II	229	COPD	71.4 (8.4)	19.6	A pulse oximeter, BP gauge, spirometer, and respiratory rate and oxygen therapy compliance monitor connected to a modem that uploaded readings to secure servers—performed daily for 12 months; alerts automatically triggered when measurements were exceeding thresholds or missing	Proportion hospitalized and length of hospital stay	12 months
Stamenova (2020)/Canada [52]	App: Cloud DX Connected Health Kit	81	COPD	72.4 (9.3)	46.0	Cloud DX Connected Health Kit: custom tablet computer, Pulse wave wrist cuff monitor (measured BP), oximeter, weighing scale and thermometer; data from all devices transmitted to a database; 3-armed study, including remote patient monitoring group and self-monitoring group: oximetry and BP daily for 6 months; when readings fell outside predetermined thresholds, clinical project specialist notified who took appropriate action	Number of hospitalizations, hospital length of stay, proportion with outpatient visits, and number of emergency visits	6 months
Temple-Oberle (2023)/Canada [53]	App: RecoverWell	72	Cancer (breast, benign ovarian or uterine mass, endometrial, or cervical)	54.90 (11.18)	100	Data input: drain outputs, surgical site photographs, and QoR15^t^ and EORTC^u^ scales; computer used by trained and designated portal monitor and patient’s surgeon to access the data and monitor patients; any red flags brought to the surgeon’s attention and prompted action either via a telephone call or an in-person appointment depending on the issue	Proportions with outpatient and emergency visits	6 weeks
Tønning (2020/2021)/Denmark [54]	Trial: RADMIS; app: Monsenso system	120	Unipolar depressive disorder	43.9 (14.2)	52.5	Multimodal monitoring and treatment based on a smartphone-based system (Monsenso system); patient monitoring of mood, sleep, and activity daily, and study nurse checking the data 3 times weekly and reacting according to the data presented	Proportion hospitalized and length of hospital stay	6 monhts
Wagenaar (2019)/Netherlands [55]	App/program: EACP^v^ including “Heartfailurematters.org” website	450	HF	66.8 (11.0)	25.8	3-arm trial: (1) EACP, (2), website heartfailurematters.org, (3) usual care; EACP group: use of the e-Vita platform with telemonitoring of weight, BP, and HR and nurse alerts if values were abnormal; website group encouraged to use the website, information leaflet, and a reminder to use the website	Proportion hospitalized	12 months
Walker (2018)/ Spain, United Kingdom, Slovenia, Estonia, and Sweden [56]	Trial: CHROMED; app: RESMON PRO DIARY using FOT, a touch-screen computer, and a mobile modem	312	COPD	71^w^	34.0	Monitoring platform used for 9 months; measurements of within-breath respiratory mechanical impedance, BP, oxygen saturation, HR, and body temperature; contact with the study nurse triggered by abnormal values	Proportion hospitalized and length of hospital stay	12 months
Wang (2023/Hong Kong [57]	App: telemedicine app and system (HealthCap)	49	Hypertension	59.9 (8.4)	53	BP readings reported by patients twice daily for a week before their consultation, with data sent to a secure computer; review of results by physicians prior to appointments; and optimal 7-day average BP and no issues, appointment deferred for 3 months; suboptimal readings or presence of symptoms, consultation attendance by patients mandatory, and doctors able to adjust medication according to guidelines	Proportion hospitalized and proportion or number with outpatient visits	6 months
Ware (2022)/Canada [58]	App: Medly smartphone app	96	T2DM, hypertension, or HF	59.0 (12.6)	44.0	Telemonitoring group: patient measurements of symptoms; alerts to the clinical team via email and a secure web portal when recorded values (weight, BP, blood glucose) exceeded target thresholds	Proportion hospitalized, number of hospitalizations, and proportions and numbers of emergency and outpatient visits	6 months
Widmer (2017)/US [59]	NR	71	Acute coronary syndrome	63.0 (10.8)	18.6	Digital health intervention involving reporting of dietary and exercise habits throughout cardiac rehabilitation and educational information for patients’ healthy lifestyles; patient-entered values in the app for BP, lipids, glucose, or weight that were out of normal range, patients asked to consult their physician	Proportion hospitalized and proportion with emergency visits	3 and 6 months
Witt Udsen (2017)/Denmark [60]	TeleCare North	1225	COPD	69.5 (9.4)	51.7	A tablet at home; information to participants about COPD management and transmission by participants of data on BP, pulse, blood oxygen saturation, and body weight to health personnel	Number of hospitalizations, length of hospital stay, numbers of outpatient and emergency visits, and use of home care services	12 months
Wood (2020)/Australia [61]	NR	60	Cystic fibrosis	31.0 (9.0)	48.7	Smartphone app with 14 questions about respiratory symptoms and well-being to be answered weekly or sooner when worsening of symptoms; if symptoms worsened, call from a nurse	Proportion hospitalized, length of hospital stay, and number of outpatient visits	12 months
Yanicelli (2021)/Argentina [62]	NR	30	HF	52.0 (NR)	20.0	Home telemonitoring system consisting of an app collecting weight, BP, and HR measurements and symptoms (eg, swelling in ankles, legs, shortness of breath); app-based alert to a physician if measurements are outside normal ranges; also included educational functionality	Proportion hospitalized	3 months

^a^NR: not reported.

^b^COPD: chronic obstructive pulmonary disease.

^c^T2DM: type 2 diabetes mellitus.

^d^HF: heart failure.

^e^BP: blood pressure.

^f^ASICA: Achieving Self-directed Integrated Cancer Aftercare.

^g^HR: heart rate.

^h^SSE: skin self‐examination.

^i^ECG: electrocardiogram.

^j^Median (range).

^k^INH: Interdisciplinary Network Heart Failure.

^l^HC: health coach.

^m^eICE: early Intervention in Cystic Fibrosis Exacerbation.

^n^CFRSD: Cystic Fibrosis Respiratory Symptom Diary.

^o^FEV1: forced expiratory volume in one second.

^p^GSM: Global System for Mobile Communications.

^q^TCC: TeleClinical Care.

^r^NSSMP: nurse-led smartphone-based self-management program.

^s^Median (IQR).

^t^QoR15: quality of recovery questionnaire.

^u^EORTC: European Organisation for Research and Treatment of Cancer.

^v^EACP: eHealth adjusted care pathway.

^w^Median.

For one study by Abelsen et al [64], we were unable to calculate effect estimates. As a result, the study was not included in the meta-analyses, and we did not assess our confidence in the results. The findings from this study are described narratively. Table 2 shows the main characteristics of the included studies. Some of the trials had specific names, and some utilized a specific app, which are listed in the second column. The interventions were mostly similar, but we provided a detailed description of each. Although the trials had many outcomes, we included only those relevant for this review. Follow-up times ranged from 1 month to 60 months, with 37 studies having follow-up times between 3 months and 12 months (Table 3).

### Risk of Bias

**Figure 2:**
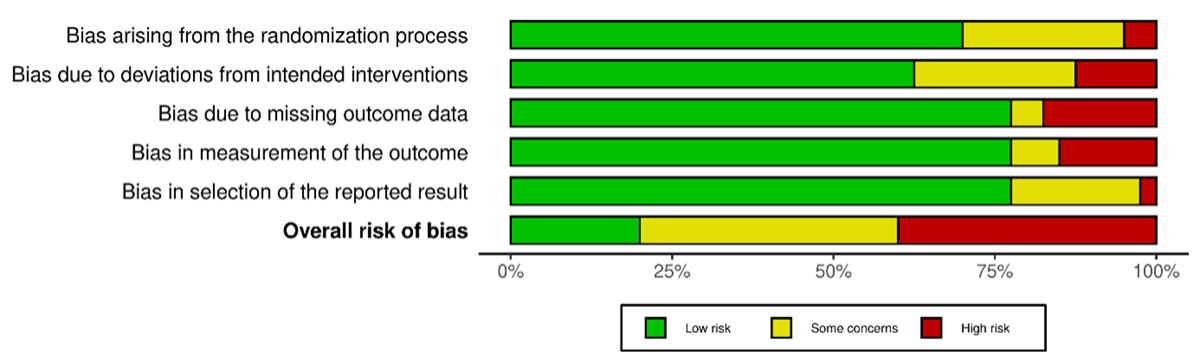
Summary risk of bias assessment.

Of 40 studies, 8 were assessed as having a low risk of bias, 16 had some concerns, and the remaining 16 were assessed as having a high risk of bias. Figure 2 shows a summary of the risk of bias assessments.

### Effects of Interventions

#### Proportion of Hospitalized Patients

**Figure 3:**
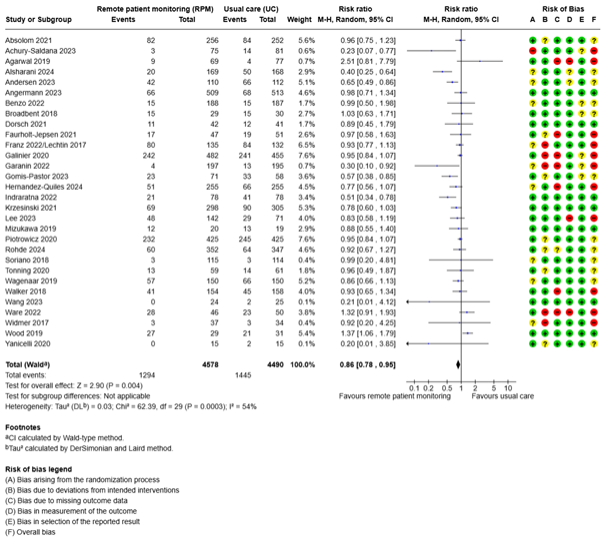
Meta-analysis of the proportion of hospitalized patients in groups randomized to remote patient monitoring or usual care, with the risk of bias assessments for the outcome shown on the right for each study.

Of the included studies, 30 reported the proportion of hospitalized patients. The RR was 0.86 (95% CI 0.77 to 0.95; low certainty; Figure 3, Multimedia Appendix 2). There was significant heterogeneity (*I*^2^: 55%). Mixed effects meta-regression analysis indicated that newer studies were more in favor of remote patient monitoring (coefficient for publication year: –0.06, *P*=.008; meaning that, for every increase of 1 publication year, the RR decreased by 0.06.) Mean age did not have a significant effect on proportion hospitalized. When both explanatory variables were entered together (full model), publication year was still significant (coefficient: –0.07, *P*=.006). In the full model, *I*^2^ was 46%. Therefore, there was still some unexplained heterogeneity. The Egger test was not significant (*P*=.07), not indicating publication bias. In the subgroup of cardiovascular disease (16 studies), the RR was 0.76 (95% CI 0.65 to 0.88). We did not use GRADE for this subgroup result. The only other diagnostic subgroup with more than 2 studies was chronic obstructive pulmonary disease (COPD; 5 studies). The RR was 0.81 (95% CI 0.66 to 1.00). For the other subgroups, there were no important differences between remote patient monitoring and usual care.

Laursen et al [45] reported the proportion with hospitalizations, but it was only reported as <5. Therefore, this could not be included in the meta-analysis. Jiang et al [43] stated that the 12-month risk of readmission was 34.3% in intervention group compared with 56.0% in the control group, but these numbers could not be included in the meta-analysis.

#### Number of Hospitalizations

**Figure 4:**
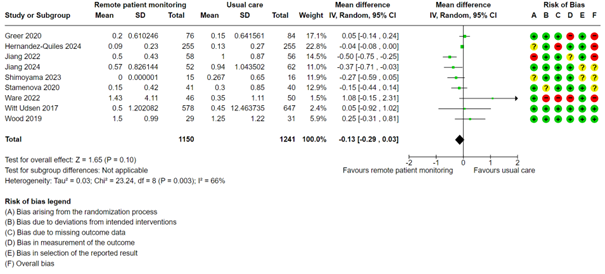
Meta-analysis of the number of hospitalizations in groups randomized to remote patient monitoring or usual care, with the risk of bias assessments for the outcome shown on the right for each study.

Patients receiving usual care had a mean number of 0.42 hospitalizations. A meta-analysis with 9 studies showed that the group with remote patient monitoring had 0.13 fewer hospitalizations (95% CI –0.29 to 0.03; low certainty; Figure 4, Multimedia Appendix 2). We did not perform mixed effects meta-regression, subgroup, or test for publication bias for this outcome.

Abelsen et al [64] measured the number of unplanned hospital admissions after 6 months and 12 months and reported no difference between the groups after 6 months. However, they found an increase in the number of admissions in the remote patient monitoring group compared with the usual care group after 12 months.

#### Length of Hospital Stay

**Figure 5:**
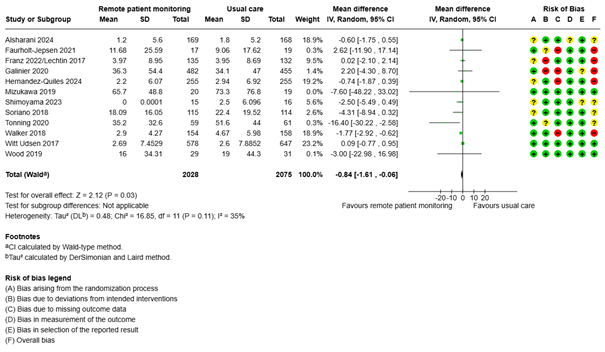
Meta-analysis of the length of hospital stay (in days) in groups randomized to remote patient monitoring or usual care, with the risk of bias assessments for the outcome shown on the right for each study.

The mean hospital length of stay in the usual care group was 6.82 days. A meta-analysis with 12 studies showed that the length of hospital stay was slightly shorter for the remote patient monitoring group, at 0.84 days shorter (95% CI –1.61 to –0.06; low certainty; Figure 5, Multimedia Appendix 2). None of the explanatory variables had a significant effect on length of hospital stay. The Egger test was not significant (*P*=.08).

Abelsen et al [63,64] measured the number of hospital bed days for unplanned admissions after 6 months and 12 months and reported no difference between the groups. Andersen et al [29] reported the median length of stay in the hospital for 0 months through 6 months as 5 days in the telegroup and 7 days in the control group. For a period of 0 months through 24 months, they reported 4 days in both groups. It was not possible to compute an effect size. Broadbent et al [32] reported the total number of days in hospital for respiratory problems in the intervention and control groups. It was not possible to compute an effect size. Garanin et al [37] reported that the intervention group was hospitalized for a mean number of 7.5 days and the usual care group was hospitalized for a mean of 10.2 days. It was not possible to compute an effect size.

#### Proportion With Outpatient Visits

**Figure 6:**
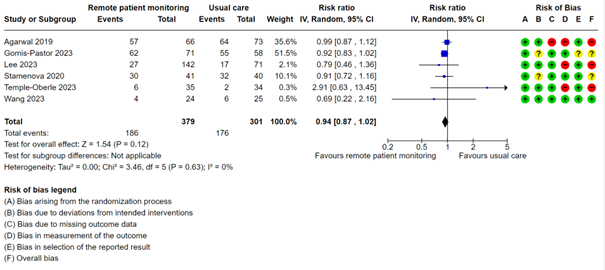
Meta-analysis of the proportion of outpatient visits in groups randomized to remote patient monitoring or usual care, with the risk of bias assessments for the outcome shown on the right for each study.

A meta-analysis with 6 studies showed almost no difference in the proportion of patients with outpatient visits between the 2 groups (RR 0.94, 95% CI 0.87 to 1.02; moderate certainty; Figure 6, Multimedia Appendix 2). We did not perform mixed effects meta-regression, subgroup based on condition, or test for publication bias for this outcome.

#### Mean Number of Outpatient Visits

**Figure 7:**
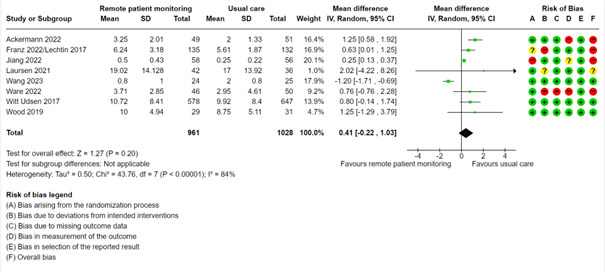
Meta-analysis of the mean number of outpatient visits in groups randomized to remote patient monitoring or usual care, with the risk of bias assessments for the outcome shown on the right for each study.

The studies came from many different countries with diverse health care systems. We decided not to distinguish between private or public clinic visits, medical consultations, private physicians, or general practitioners. Instead, we categorized all these consultations as “outpatient visits.” The usual care group had a mean number of 8.13 outpatient visits. A meta-analysis with 8 studies showed that the remote patient monitoring group had slightly more outpatient visits (0.41 more), thus favoring the usual care group, but the 95% CI crossed zero (95% CI –0.22 to 1.03; low certainty; Figure 7, Multimedia Appendix 2). We did not perform mixed effects meta-regression, subgroup based on condition, or test for publication bias for this outcome.

Abelsen et al [63,64] measured the number of outpatient visits and number of contacts with general practitioners after 6 months and 12 months. They found no difference in the number of outpatient visits and a tendency toward a greater increase in the number of general practitioner consultations in the remote patient monitoring group compared with the usual care group after 6 months and 12 months.

#### Proportion With Emergency Visits

**Figure 8:**
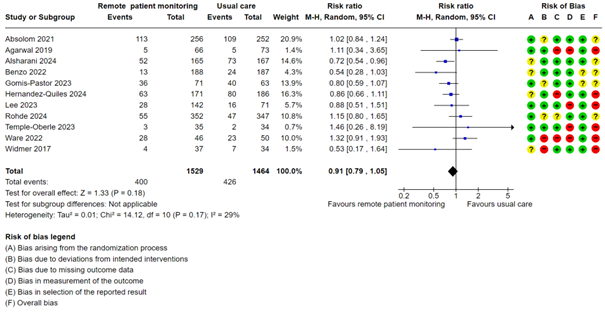
Meta-analysis on the proportion of emergency visits in groups randomized to remote patient monitoring or usual care, with the risk of bias assessments for the outcome shown on the right for each study.

A meta-analysis with 11 studies showed little or no difference between the groups on proportion of emergency visits (RR 0.91, 95% CI 0.79 to 1.05; low certainty; Figure 8, Multimedia Appendix 2). There was no indication of publication bias (Egger test, *P*=.75). We did not perform mixed effects meta-regression or subgroup analysis based on condition because the heterogeneity was low.

#### Number of Emergency Visits

**Figure 9:**
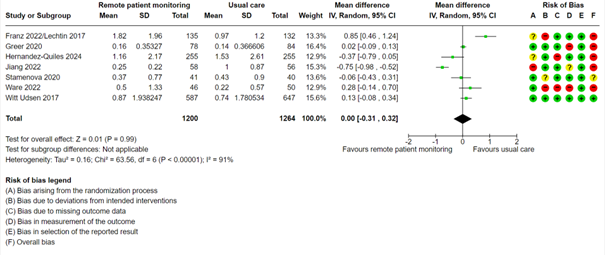
Meta-analysis on the number of emergency visits in groups randomized to remote patient monitoring or usual care, with the risk of bias assessments for the outcome shown on the right for each study.

The usual care group had a mean number of 0.86 emergency visits. A meta-analysis with 7 studies showed very low certainty evidence for the number of emergency visits (mean difference 0.00, 95% CI –0.31 to 0.32; very low certainty; Figure 9, Multimedia Appendix 2). We did not perform mixed effects meta-regression, perform subgroup analysis based on condition, or test for publication bias.

Abelsen et al [63,64] examined both the number of stays in municipal acute care and the number of contacts with emergency services after 6 months and 12 months. The study found no differences between the groups for either measure.

#### Home Care Services

Witt Udsen 2017 [60] measured the average hours spent on health care services at home over a 12- month period, finding that more hours were spent on home health care services in the remote patient monitoring group compared with the usual care group (mean difference 523.2, 95% CI –152.6 to 1199.0 hours).

Similarly, Abelsen et al [63,64] measured the average number of home health care visits after 6 months and 12 months and reported no differences between groups.

#### Institutional Stay

None of the included studies reported on institutional stay, only hospitalizations.

#### Absolute Effects

So far, we presented relative effects of remote patient monitoring compared with usual care, reporting RRs and mean differences. However, to fully grasp the impact and understand how many individuals are actually affected, it is important to also consider the absolute numbers. Table 4 shows the proportions of hospitalizations, outpatient visits, and emergency visits for the 2 groups, and Table 5 shows the numbers of hospitalizations, outpatient visits, and emergency visits and length of hospital stay in days.

**Table 4:** Proportions with hospitalizations, outpatient visits, and emergency visits in remote patient monitoring versus usual care.

Outcomes	Usual care	Remote patient monitoring
Proportion hospitalized	0.32	0.28
Proportion with outpatient visits	0.59	0.55
Proportion with emergency visits	0.29	0.26

**Table 5:** Absolute numbers of hospitalizations, days in hospital, outpatient visits, and emergency visits in remote patient monitoring versus usual care.

Outcomes	Usual care	Remote patient monitoring
Number of hospitalizations	0.42	0.29
Length of hospital stay	6.82	6.01
Number of outpatient visits	8.13	8.54
Number of emergency visits	0.86	0.86

## Discussion

### Principal Findings

Remote patient monitoring may slightly reduce the proportion of hospital stays, but it may make little or no difference to the number or length of hospital stays. It likely has little or no effect on the proportion of patients with outpatient visits and may result in little or no difference in the mean number of outpatient visits or the proportion with emergency visits. The evidence is uncertain regarding the effect of remote patient monitoring on the number of emergency visits.

Other systematic reviews have similar findings. Iqbal and colleagues [65], who examined clinical outcomes of digital alerting systems in remote monitoring, also found reductions in hospitalizations and length of stay for certain cohorts. However, they found no important benefit on emergency department or outpatient visits. Similarly, a review by Auener et al [66] on telemonitoring for chronic heart failure patients showed no clear differences in health care utilization between the remote monitoring group and usual care, except for an increase in nonemergency outpatient department visits for the remote monitoring group. These findings suggest that closer monitoring through remote patient monitoring may lead to more frequent health care interactions in an outpatient setting. Improved condition management through continuous monitoring and timely intervention can potentially lower the incidence of critical health episodes, reducing the demand for more costly interventions like hospitalizations. Patients who are remotely monitored may also feel a greater sense of security or may be more inclined to seek medical attention for minor symptoms or concerns that they might otherwise have ignored. This increased health care–seeking behavior could lead to higher health care utilization rates of, for example, outpatient clinical visits and potentially counteract savings from remote monitoring.

Hospitalizations are highly resource-intensive and costly [67]. Although our findings on the absolute reduction in the proportion of hospitalized patients may seem small, with a reduction from 32% to 28%, this modest reduction is expected to result in significant cost savings. Fewer hospitalizations and shorter hospital stays can lead to decreased health care costs per patient and allow for more efficient use of hospital resources, such as freeing up beds and reducing the burden on health care staff. This, in turn, enables hospitals to accommodate more patients and potentially reduces wait times for critical care.

Previous reviews have shown mixed results regarding the cost-effectiveness of remote patient monitoring [68], and remote patient monitoring systems sometimes lead to increased utilization of health care services rather than the anticipated reduction. The effectiveness of remote monitoring in reducing health care resource utilization may vary depending on the specific patient population, disease condition, and health care setting [69]. Thomas et al [14] identified several factors contributing to increased health care utilization. These included recruiting high-risk populations, failure to integrate remote patient monitoring into workflows, and system measurement errors. Specific issues such as slow responses to alerts and low adherence to the remote follow-up by patients or clinicians were significant contributors to increased acute care use in the remote patient monitoring group. To reduce hospitalizations, it is crucial that remote patient monitoring systems accurately predict disease exacerbations by detecting changes in symptoms related to disease worsening. This can be particularly challenging in populations with unpredictable disease progression [14]. Patients who are more prone to frequent hospitalizations may experience a greater reduction in admissions with timely interventions made possible by remote monitoring. However, in practice, clinicians may hesitate to rely on remote assessments for their most vulnerable and critically ill patients [14]. The subgroup analysis in our review indicated that remote patient monitoring may reduce hospitalizations for patients with heart disease and COPD, but among the other NCDs, there were little or no differences. A systematic review of evaluations of noninvasive remote patient monitoring for chronic diseases [69] found that remote patient monitoring was highly cost-effective for hypertension. However, for COPD and heart failure, the cost-effectiveness varied depending on the severity of the disease. These findings suggest that certain patient populations may benefit more from remote monitoring than others and that variations in outcomes are influenced by factors such as diagnosis severity, health care setting, and the specific design of the remote patient monitoring intervention. Consequently, the impact on health care resource utilization may differ across contexts.

Regarding organizational aspects, remote patient monitoring can potentially streamline care, but it can also introduce complexities that may offset resource savings. For instance, remote patient monitoring generates large volumes of health data, which must be processed, cleaned, analyzed, and managed before it can be reliably used for clinical decision support. This not only requires robust data handling procedures but also the allocation of resources and adaptation of existing health care infrastructure to effectively integrate these systems into routine care [70,71]. Moreover, following up missing data due to lack of patient adherence or problems with devices presents additional, resource-intensive challenges that must be addressed to ensure safe and equitable care [72]. Alignment and adaptation of infrastructure, along with training and upskilling of staff, seem crucial to meet the demands of remote monitoring, ensuring continuity of patient care without creating gaps. Substantial investments in technology, training, and support are required to implement remote patient monitoring, and these costs may not be immediately offset by savings [69]. There is also a rapid advancement in digital solutions, and our results show that newer studies suggest better results in terms of resource utilization than older studies.

### Strengths and Limitations

This systematic review’s selection of eligible studies relied on a clearly defined and systematic search strategy. Although it is possible that we overlooked studies due to insufficient descriptions of interventions or populations in the title or abstract, we believe it is likely that we identified the most relevant studies. We only included studies in which the intervention was conducted in 2013 or later. This may have excluded valuable research from earlier periods. However, given the significant advancements in technology in recent years, we prioritized newer studies to provide more realistic and up-to-date results. We did not examine health care personnels’ resource use related to remote follow-up, though previous studies suggest that handling incoming data from patient-reported outcome measures can be time-consuming for staff and may place a significant burden on health care providers in terms of time and resources [73].

Further, we assessed the risk of bias and certainty of the evidence transparently and systematically. These assessments allow readers to make their own judgments of the strength of the evidence. However, in many of the studies, the settings, interventions, and populations were poorly described, which may introduce limitations when synthesizing results from studies with different populations and intervention types. We attempted to account for this in our GRADE assessments, but due to poor reporting and a lack of detailed information about the settings, there may still be challenges related to heterogeneity. This could affect the overall interpretation of the findings and reduce confidence in the conclusions drawn. Moreover, regarding the outcome of outpatient visits, it is somewhat unclear what specific terms like “outpatient clinic visits,” “outpatient visits,” “medical consultations,” and visits to “private physicians” or “general practitioners” precisely mean in the studies we reviewed. This inconsistency in terminology represents a limitation and should be considered carefully when interpreting this outcome, as it may affect the comparability of health care utilization results across the studies.

The evidence base is heavily skewed toward studies conducted in high-income countries, which limits the applicability of the findings to low- and middle-income settings. Additionally, considerable heterogeneity in patient populations and interventions across studies complicates the ability to draw broader, generalizable conclusions. The lack of economic evaluations in this review also limits our ability to assess cost-effectiveness, which is a critical factor for informing health care policy and implementation decisions.

### Implications for Future Research

Future research should explore ways to streamline the processing of data from patient-reported outcomes measures through automated tools and improved system integration to optimize efficiency. Understanding the relationship between remote monitoring and resource utilization—along with identifying barriers and facilitators—is crucial to develop services that can maintain or improve current standards of care while being more resource-efficient. There is also a need for systematic reviews that examine more homogeneous patient groups and forms of digital home follow-up to better understand which groups may benefit from specific treatments. Additionally, a more comprehensive health-economic analysis is needed, considering resource use more holistically, including costs associated with technology acquisition, maintenance, follow-up on incoming data, service usage, and impacts on work absence, among other factors.

### Conclusions

This systematic review showed that remote patient monitoring possibly led to lower proportions of patients being hospitalized, fewer hospitalizations, and shorter hospital length of stay compared with usual care. The proportion of patients with outpatient visits was probably similar for remote patient monitoring and usual care. Remote patient monitoring had possibly more outpatient visits compared with usual care. The proportion with emergency visits was possibly similar between the groups. We are uncertain about the effect on number of emergency visits. The results should be considered with caution as the certainty of evidence was moderate to very low. We did not find results regarding institutional stay.

## Appendix

Multimedia Appendix 1 PRISMA checklist.

Multimedia Appendix 2 Search strategy and Grading of Recommendations Assessment, Development, and Evaluation (GRADE) assessments.

## Abbreviations

COPD: chronic obstructive pulmonary disease
GRADE: Grading of Recommendations Assessment, Development, and Evaluation
GSM: Global System for Mobile Communications module
NCD: noncommunicable disease
PRISMA: Preferred Reporting Items for Systematic Reviews and Meta-Analyses
RoB 2: Cochrane risk-of-bias Tool
RR: risk ratio
