# Supplementary material for: Effects of Remote Patient Monitoring on Health Care Utilization in Patients With Noncommunicable Diseases: Systematic Review and Meta-Analysis
Source: JMIR Mhealth Uhealth. 2025 Oct 1;13:e68464. doi: 10.2196/68464 (PMC12530163; doi:10.2196/68464)
Supplement: Multimedia Appendix 2 [file mhealth_v13i1e68464_app2.pdf]

Appendix 1: search strategy

First search date: 2022-04-26

Database: Ovid MEDLINE(R) and Epub Ahead of Print, In-Process, In-Data-Review & Other Non-Indexed Citations, Daily and Versions <1946 to April 22, 2022> Search date: 2022-04-26

1 (tele* medicine* or telemedicine or tele* nurs* or telenurs* or tele* support* or telesupport* or tele* mental* or telemental* or telecare* or tele* care* or teleconsult* or tele* consult* or telefollow* or tele* follow* or telehealth* or tele* health* or telehome* or tele* home* or telemanag* or tele*manage* or telepatien* or tele* patient* or telesupport* or tele* support* or e-care* or ecare* or e-health* or ehealth* or e-medicine* or emedicine* or e-nursing* or enursing* or mnurs* or e-consult* or econsult* or e-mental* or emental* or mcare or mconsult* or m consult* or mhealth or mmedicine or mmental* or video conference* or videoconference* or video consult* or videoconsult* or iphone* or ipad* or smart device* or e-mail* or email* or ((digital* or tele* or remote* or distant*) adj3 (care* or healthcare* or consult* or observ* or check or checking or checkup* or treatment* or therap*)) or ((digital* or remote or distant or distance*) adj2 patient*) or ((text or phone) adj messaging) or ((mobile or smartphone or tablet) adj3 (app or apps or application*))).ti,ab,kf. (109092)

2 (home adj2 (monitoring* or technolog*)).ti,ab,kf. (4226)

3 Telemedicine/ or Telerehabilitation/ or Remote Consultation/ or Telenursing/ or Videoconferencing/ or Telecommunications/ or Electronic Mail/ or Telephone/ or Cell Phone/ or Smartphone/ or Text Messaging/ or Mobile Applications/ (79150)

4 or/1-3 (153275)

5 exp Cardiovascular Diseases/ (2609661)

6 (cardio* or cardia* or heart* or coronary* or angina* or ventric* or myocard* or pericard* or isch?m* or emboli* or arrhythmi* or thrombo* or atrial fibrillat* or tachycardi* or endocardi* or sick sinus or hypertension* or stroke or ((vascular or cerebrovascular or arterial) adj (disease* or disorder*)) or ventricular).ti,ab,kf. (3300365)

7 Noncommunicable Diseases/ (2353)

8 ((non-communicable or non-infectious or noncommunicable or noninfectious or chronic) adj disease*).ti,ab,kf. (91969)

9 exp Lung Diseases/ (1107816)

10 (((lung* or pulmonary) adj3 disease*) or cystic fibrosis or asthma* or sarcoidos*).ti,ab,kf. (382080)

11 exp Neoplasms/ (3673697)

12 (cancer* or neoplasm*).ti,ab,kf. (2281894)

13 exp Diabetes Mellitus/ (477037)

14 (diabetes or diabetic or hyperglycemia* or glucose intolerance).ti,ab,kf. [sjekk staving] (730800)

15 exp Mental Disorders/ (1367384)

16 (eating disorder* or anorexia nervosa or bulimi* or binge eat* or (self adj (injur* or mutilat*)) or suicide* or suicidal or parasuicid* or mood disorder* or affective disorder* or bipolar or mania or manic or depression or depressive or dysthymi* or neurotic or neurosis or adjustment disorder* or anxiety disorder* or obsess* or compulsi* or panic or phobi* or ptsd or posttrauma* or post trauma* or chronic fatigue* or affective symptoms or mental disorder* or mental health or schizo* or (mental* adj2 ill*)).ti,ab,kf. (1015462)

17 or/5-16 (10895508)

18 4 and 17 (64569)

19 randomized controlled trial.pt. (566020)

20 controlled clinical trial.pt. (94836)

21 (randomized or randomised).ti,ab. (721501)

22 randomly.ab. (380633)

23 (randomis* or randomiz* or randomly).ti,ab. (1033166)

24 groups.ab. (2340038)

25 (trial or study).ti. (1813276)

26 or/19-25 (4454606)

27 exp Animals/ (25408164)

28 Humans/ (20410303)

29 27 not (27 and 28) (4997861)

30 review.pt. (2972785)

31 meta analysis.pt. (158657)

32 news.pt. (212099)

33 comment.pt. (960770)

34 editorial.pt. (602416)

35 cochrane database of systematic reviews.jn. (15803)

36 comment on.cm. (960717)

37 (systematic review or literature review).ti. (226080)

38 or/29-37 (9454069)

39 26 not 38 [tilpasset cochrane sensitive rct] (3534576)

40 18 and 39 (20036)

41 limit 18 to "therapy (maximizes sensitivity)" (20579)

42 limit 41 to yr="2022 -Current" (821)

43 limit 40 to yr="2022 -Current" (1238)

44 42 or 43 (1506)

Database: Embase <1974 to 2022 April 22> Search date: 2002-04-26

1 (tele* medicine* or telemedicine or tele* nurs* or telenurs* or tele* support* or telesupport* or tele* mental* or telemental* or telecare* or tele* care* or teleconsult* or tele* consult* or telefollow* or tele* follow* or telehealth* or tele* health* or telehome* or tele* home* or telemanag* or tele*manage* or telepatien* or tele* patient* or telesupport* or tele* support* or e-care* or ecare* or e-health* or ehealth* or e-medicine* or emedicine* or e-nursing* or enursing* or mnurs* or e-consult* or econsult* or e-mental* or emental* or mcare or mconsult* or m consult* or mhealth or mmedicine or mmental* or video conference* or videoconference* or video consult* or videoconsult* or iphone* or ipad* or smart device* or e-mail* or email* or ((digital* or tele* or remote* or distant*) adj3 (care* or healthcare* or consult* or observ* or check or checking or checkup* or treatment* or therap*)) or ((digital* or remote or distant or distance*) adj2 patient*) or ((text or phone) adj messaging) or ((mobile or smartphone or tablet) adj3 (app or apps or application*))).ti,ab,kf. (159331)

2 (home adj2 (monitoring* or technolog*)).ti,ab,kf. (6332)

3 *telemedicine/ or *telerehabilitation/ or *teleconsultation/ or *telenursing/ or *videoconferencing/ or *telecommunication/ or *e-mail/ or *telephone/ or *mobile phone/ or *smartphone/ or *text messaging/ or *mobile application/ (66159)

4 or/1-3 (195292)

5 exp *cardiovascular disease/ (2717037)

6 (cardio* or cardia* or heart* or coronary* or angina* or ventric* or myocard* or pericard* or isch?m* or emboli* or arrhythmi* or thrombo* or atrial fibrillat* or

tachycardi* or endocardi* or sick sinus or hypertension* or stroke or ((vascular or cerebrovascular or arterial) adj (disease* or disorder*)) or ventricular).ti,ab,kf. (4478857)

7 *non communicable disease/ or exp *chronic disease/ (35824)

8 ((non-communicable or non-infectious or noncommunicable or noninfectious or chronic) adj disease*).ti,ab,kf. (125323)

9 exp *lung disease/ (949407)

10 (((lung* or pulmonary) adj3 disease*) or cystic fibrosis or asthma* or sarcoidos*).ti,ab,kf. (546328)

11 exp *malignant neoplasm/ (2835235)

12 (cancer* or neoplasm*).ti,ab,kf. (3105983)

13 exp *diabetes mellitus/ (546390)

14 (diabetes or diabetic or hyperglycemia* or glucose intolerance).ti,ab,kf. [sjekk staving] (1089136)

15 exp *mental disease/ (1479871)

16 (eating disorder* or anorexia nervosa or bulimi* or binge eat* or (self adj (injur* or mutilat*)) or suicide* or suicidal or parasuicid* or mood disorder* or affective disorder* or bipolar or mania or manic or depression or depressive or dysthymi* or neurotic or neurosis or adjustment disorder* or anxiety disorder* or obsess* or compulsi* or panic or phobi* or ptsd or posttrauma* or post trauma* or chronic fatigue* or affective symptoms or mental disorder* or mental health or schizo* or (mental* adj2 ill*)).ti,ab,kf. (1302483)

17 or/5-16 (12342782)

18 4 and 17 (79348)

19 exp randomized controlled trial/ (707101)

20 (randomized or randomised).ti,ab. (1035251)

21 randomly.ab. (504258)

22 groups.ab. (3262929)

23 (trial or study).ti. (2210385)

24 or/19-23 (5760138)

25 18 and 24 (26541)

26 limit 25 to yr="2022 -Current" (1140)

27 limit 26 to embase (671)

Database: Cochrane Central Register of Controlled Trials Search date: 2022-04-26

#1 ((tele* NEXTmedicine*) or telemedicine or (tele* NEXT nurs*) or telenurs* or (tele* NEXT support*) or telesupport* or (tele* NEXT mental*) or telemental* or telecare* or (tele* NEXT care*) or (tele NEXT consult*) or teleconsult* or telefollow* or (tele* NEXT follow*) or telehealth* or (tele* NEXT health*) or telehome* or (tele* NEXT home*) or telemanag* or (tele NEXT manage*) or telepatien* or (tele* NEXT patient*) or telesupport* or (tele* NEXT support*) or e-care* or ecare* or e-health* or ehealth* or e-medicine* or emedicine* or e-nursing* or enursing* or mnurs* or e-consult* or econsult* or e-mental* or emental* or mcare or mconsult* or m-consult* or mhealth or mmedicine or mmental* or video-conference* or videoconference* or video-consult* or videoconsult* or iphone* or ipad* or smart-device* or e-mail* or email* or ((digital* or tele* or remote* or distant*) NEAR/3 (care* or healthcare* or consult* or observ* or check or checking or checkup* or treatment* or therap*)) or ((digital* or remote or distant or distance*) NEAR/2 patient*) or ((text or phone) NEXT messaging) or ((mobile or smartphone or tablet) NEAR/2 (app or apps or application*))):ti,ab,kw 49197

#2 (home NEAR/2 (monitoring* or technolog*)) 1241

#3 MeSH descriptor: [Telemedicine] this term only 2657

#4 MeSH descriptor: [Telerehabilitation] this term only 166

#5 MeSH descriptor: [Remote Consultation] this term only 386

#6 MeSH descriptor: [Telenursing] this term only 31

#7 MeSH descriptor: [Videoconferencing] this term only 218

#8 MeSH descriptor: [Telecommunications] this term only 89

#9 MeSH descriptor: [Electronic Mail] this term only 358

#10 MeSH descriptor: [Telephone] this term only 2319

#11 MeSH descriptor: [Cell Phone] this term only 776

#12 MeSH descriptor: [Smartphone] this term only 621

#13 MeSH descriptor: [Text Messaging] this term only 1101

#14 MeSH descriptor: [Mobile Applications] this term only 1020

#15 #1 OR #2 OR #3 OR #4 OR #5 OR #6 OR #7 OR #8 OR #9 OR #10 OR #11 OR #12 OR #13 OR #14 50361

#16 MeSH descriptor: [Cardiovascular Diseases] explode all trees 116028

#17 (cardio* or cardia* or heart* or coronary* or angina* or ventric* or myocard* or pericard* or ischem* or ischaem* or emboli* or arrhythmi* or thrombo* or (atrial NEXT fibrillat*) or tachycardi* or endocardi* or sick-sinus or hypertension* or stroke or ((vascular or cerebrovascular or arterial) NEXT (disease* or disorder*)) or ventricular):ti,ab,kw 388507

#18 MeSH descriptor: [Noncommunicable Diseases] this term only 44

#19 ((non-communicable or non-infectious or noncommunicable or noninfectious or chronic) NEXT disease*):ti,ab,kw 23772

#20 MeSH descriptor: [Lung Diseases] explode all trees 45069

#21 (((lung* or pulmonary) NEAR/3 disease*) or cystic-fibrosis or asthma* or sarcoidos*):ti,ab,kw 65709

#22 MeSH descriptor: [Neoplasms] explode all trees 87370

#23 (cancer* or neoplasm*):ti,ab,kw 200394

#24 MeSH descriptor: [Diabetes Mellitus] explode all trees 34672

#25 (diabetes or diabetic or hyperglycemia* or glucose-intolerance):ti,ab,kw 106655

#26 MeSH descriptor: [Mental Disorders] explode all trees 80252

#27 (eating-disorder* or anorexia-nervosa or bulimi* or binge-eat* or (self NEXT (injur* or mutilat*)) or suicide* or suicidal or parasuicid* or mood-disorder* or affective-disorder* or bipolar or mania or manic or depression or depressive or dysthymi* or neurotic or neurosis or adjustment-disorder* or anxiety-disorder* or obsess* or compulsi* or panic or phobi* or ptsd or posttrauma* or post-trauma* or chronic-fatigue* or affective-symptoms or mental-disorder* or mental-health or schizo* or (mental* NEAR/2 ill*)):ti,ab,kw 152395

#28 #16 or #17 or #18 or #19 or #20 or #21 or #22 or #23 or #24 or #25 or #26 or #27 866324

#29 #15 and #28 with Publication Year from 2017 to 2022, in Trials 14351

Search updated: 2023-05-08

Database: Ovid MEDLINE(R) and Epub Ahead of Print, In-Process, In-Data-Review & Other Non-Indexed Citations, Daily and Versions <1946 to May 07, 2023> Search date: 2023-05-08

1 (tele* medicine* or telemedicine or tele* nurs* or telenurs* or tele* support* or telesupport* or tele* mental* or telemental* or telecare* or tele* care* or teleconsult* or tele* consult* or telefollow* or tele* follow* or telehealth* or tele* health* or telehome* or tele* home* or telemanag* or tele*manage* or telepatien* or tele*

patient* or telesupport* or tele* support* or e-care* or ecare* or e-health* or ehealth* or e-medicine* or emedicine* or e-nursing* or enursing* or mnurs* or e-consult* or econsult* or e-mental* or emental* or mcare or mconsult* or m consult* or mhealth or mmedicine or mmental* or video conference* or videoconference* or video consult* or videoconsult* or iphone* or ipad* or smart device* or e-mail* or email* or ((digital* or tele* or remote* or distant*) adj3 (care* or healthcare* or consult* or observ* or check or checking or checkup* or treatment* or therap*)) or ((digital* or remote or distant or distance*) adj2 patient*) or ((text or phone) adj messaging) or ((mobile or smartphone or tablet) adj3 (app or apps or application*))).ti,ab,kf. (126726)

2 (home adj2 (monitoring* or technolog*)).ti,ab,kf. (4649)

3 Telemedicine/ or Telerehabilitation/ or Remote Consultation/ or Telenursing/ or Videoconferencing/ or Telecommunications/ or Electronic Mail/ or Telephone/ or Cell Phone/ or Smartphone/ or Text Messaging/ or Mobile Applications/ (86303)

4 or/1-3 (173212)

5 exp Cardiovascular Diseases/ (2702463)

6 (cardio* or cardia* or heart* or coronary* or angina* or ventric* or myocard* or pericard* or isch?m* or emboli* or arrhythmi* or thrombo* or atrial fibrillat* or tachycardi* or endocardi* or sick sinus or hypertension* or stroke or ((vascular or cerebrovascular or arterial) adj (disease* or disorder*)) or ventricular).ti,ab,kf. (3472480)

7 Noncommunicable Diseases/ (2979)

8 ((non-communicable or non-infectious or noncommunicable or noninfectious or chronic) adj disease*).ti,ab,kf. (102028)

9 exp Lung Diseases/ (1207109)

10 (((lung* or pulmonary) adj3 disease*) or cystic fibrosis or asthma* or sarcoidos*).ti,ab,kf. (402145)

11 exp Neoplasms/ (3826287)

12 (cancer* or neoplasm*).ti,ab,kf. (2450339)

13 exp Diabetes Mellitus/ (502989)

14 (diabetes or diabetic or hyperglycemia* or glucose intolerance).ti,ab,kf. [sjekk staving] (782428)

15 exp Mental Disorders/ (1424351)

16 (eating disorder* or anorexia nervosa or bulimi* or binge eat* or (self adj (injur* or mutilat*)) or suicide* or suicidal or parasuicid* or mood disorder* or affective disorder* or bipolar or mania or manic or depression or depressive or dysthymi* or neurotic or

neurosis or adjustment disorder* or anxiety disorder* or obsess* or compulsi* or panic or phobi* or ptsd or posttrauma* or post trauma* or chronic fatigue* or affective symptoms or mental disorder* or mental health or schizo* or (mental* adj2 ill*)).ti,ab,kf. (1089961)

17 or/5-16 (11484033)

18 4 and 17 (74723)

19 randomized controlled trial.pt. (592094)

20 controlled clinical trial.pt. (95285)

21 (randomized or randomised).ti,ab. (778518)

22 randomly.ab. (407688)

23 (randomis* or randomiz* or randomly).ti,ab. (1111752)

24 groups.ab. (2512817)

25 (trial or study).ti. (1966940)

26 or/19-25 (4779373)

27 exp Animals/ (26345917)

28 Humans/ (21227240)

29 27 not (27 and 28) (5118677)

30 review.pt. (3146563)

31 meta analysis.pt. (180330)

32 news.pt. (218785)

33 comment.pt. (1006001)

34 editorial.pt. (648454)

35 cochrane database of systematic reviews.jn. (16242)

36 comment on.cm. (1005945)

37 (systematic review or literature review).ti. (267997)

38 or/29-37 (9850574)

39 26 not 38 [tilpasset cochrane sensitive rct] (3810158)

40 18 and 39 (23762)

41 limit 18 to "therapy (maximizes sensitivity)" (23060)

42 limit 41 to yr="2023 -Current" (955)

43 limit 40 to yr="2023 -Current" (1405)

44 42 or 43 (1712)

Database: Embase <1974 to 2023 May 08> Search date: 2023-05-08

1 (tele* medicine* or telemedicine or tele* nurs* or telenurs* or tele* support* or telesupport* or tele* mental* or telemental* or telecare* or tele* care* or teleconsult* or tele* consult* or telefollow* or tele* follow* or telehealth* or tele* health* or telehome* or tele* home* or telemanag* or tele*manage* or telepatien* or tele* patient* or telesupport* or tele* support* or e-care* or ecare* or e-health* or ehealth* or e-medicine* or emedicine* or e-nursing* or enursing* or mnurs* or e-consult* or econsult* or e-mental* or emental* or mcare or mconsult* or m consult* or mhealth or mmedicine or mmental* or video conference* or videoconference* or video consult* or videoconsult* or iphone* or ipad* or smart device* or e-mail* or email* or ((digital* or tele* or remote* or distant*) adj3 (care* or healthcare* or consult* or observ* or check or checking or checkup* or treatment* or therap*)) or ((digital* or remote or distant or distance*) adj2 patient*) or ((text or phone) adj messaging) or ((mobile or smartphone or tablet) adj3 (app or apps or application*))).ti,ab,kf. (188504)

2 (home adj2 (monitoring* or technolog*)).ti,ab,kf. (7149)

3 *telemedicine/ or *telerehabilitation/ or *teleconsultation/ or *telenursing/ or *videoconferencing/ or *telecommunication/ or *e-mail/ or *telephone/ or *mobile phone/ or *smartphone/ or *text messaging/ or *mobile application/ (76006)

4 or/1-3 (227962)

5 exp *cardiovascular disease/ (2947896)

6 (cardio* or cardia* or heart* or coronary* or angina* or ventric* or myocard* or pericard* or isch?m* or emboli* or arrhythmi* or thrombo* or atrial fibrillat* or tachycardi* or endocardi* or sick sinus or hypertension* or stroke or ((vascular or cerebrovascular or arterial) adj (disease* or disorder*)) or ventricular).ti,ab,kf. (4830371)

7 *non communicable disease/ or exp *chronic disease/ (40516)

8 ((non-communicable or non-infectious or noncommunicable or noninfectious or chronic) adj disease*).ti,ab,kf. (140548)

9 exp *lung disease/ (1028428)

10 (((lung* or pulmonary) adj3 disease*) or cystic fibrosis or asthma* or sarcoidos*).ti,ab,kf. (590757)

11 exp *malignant neoplasm/ (2790407)

12 (cancer* or neoplasm*).ti,ab,kf. (3411792)

13 exp *diabetes mellitus/ (596998)

14 (diabetes or diabetic or hyperglycemia* or glucose intolerance).ti,ab,kf. [sjekk staving] (1195791)

15 exp *mental disease/ (1583960)

16 (eating disorder* or anorexia nervosa or bulimi* or binge eat* or (self adj (injur* or mutilat*)) or suicide* or suicidal or parasuicid* or mood disorder* or affective disorder* or bipolar or mania or manic or depression or depressive or dysthymi* or neurotic or neurosis or adjustment disorder* or anxiety disorder* or obsess* or compulsi* or panic or phobi* or ptsd or posttrauma* or post trauma* or chronic fatigue* or affective symptoms or mental disorder* or mental health or schizo* or (mental* adj2 ill*)).ti,ab,kf. (1424123)

17 or/5-16 (13311108)

18 4 and 17 (93238)

19 exp randomized controlled trial/ (784255)

20 (randomized or randomised).ti,ab. (1143413)

21 randomly.ab. (548072)

22 groups.ab. (3558664)

23 (trial or study).ti. (2461026)

24 or/19-23 (6310246)

25 18 and 24 (31975)

26 limit 25 to yr="2023 -Current" (1422)

27 limit 26 to embase (770)

Database: Cochrane Central Register of Controlled Trials Search date: 2023-05-08

#1 ((tele* NEXTmedicine*) or telemedicine or (tele* NEXT nurs*) or telenurs* or (tele* NEXT support*) or telesupport* or (tele* NEXT mental*) or telemental* or telecare* or (tele* NEXT care*) or (tele NEXT consult*) or teleconsult* or telefollow* or (tele* NEXT follow*) or telehealth* or (tele* NEXT health*) or telehome* or (tele* NEXT home*) or telemanag* or (tele NEXT manage*) or telepatien* or (tele* NEXT patient*) or telesupport* or (tele* NEXT support*) or e-care* or ecare* or e-health* or ehealth* or e-medicine* or emedicine* or e-nursing* or enursing* or mnurs* or e-consult* or econsult* or e-mental* or emental* or mcare or mconsult* or m-consult* or mhealth or mmedicine or mmental* or video-conference* or videoconference* or video-consult* or

videoconsult* or iphone* or ipad* or smart-device* or e-mail* or email* or ((digital* or tele* or remote* or distant*) NEAR/3 (care* or healthcare* or consult* or observ* or check or checking or checkup* or treatment* or therap*)) or ((digital* or remote or distant or distance*) NEAR/2 patient*) or ((text or phone) NEXT messaging) or ((mobile or smartphone or tablet) NEAR/2 (app or apps or application*))):ti,ab,kw 57308

#2 (home NEAR/2 (monitoring* or technolog*)) 1352

#3 MeSH descriptor: [Telemedicine] this term only 3522

#4 MeSH descriptor: [Telerehabilitation] this term only 272

#5 MeSH descriptor: [Remote Consultation] this term only 415

#6 MeSH descriptor: [Telenursing] this term only 46

#7 MeSH descriptor: [Videoconferencing] this term only 330

#8 MeSH descriptor: [Telecommunications] this term only 106

#9 MeSH descriptor: [Electronic Mail] this term only 406

#10 MeSH descriptor: [Telephone] this term only 3074

#11 MeSH descriptor: [Cell Phone] this term only 918

#12 MeSH descriptor: [Smartphone] this term only 1006

#13 MeSH descriptor: [Text Messaging] this term only 1483

#14 MeSH descriptor: [Mobile Applications] this term only 1538

#15 #1 OR #2 OR #3 OR #4 OR #5 OR #6 OR #7 OR #8 OR #9 OR #10 OR #11 OR #12 OR #13 OR #14 58703

#16 MeSH descriptor: [Cardiovascular Diseases] explode all trees 150105

#17 (cardio* or cardia* or heart* or coronary* or angina* or ventric* or myocard* or pericard* or ischem* or ischaem* or emboli* or arrhythmi* or thrombo* or (atrial NEXT fibrillat*) or tachycardi* or endocardi* or sick-sinus or hypertension* or stroke or ((vascular or cerebrovascular or arterial) NEXT (disease* or disorder*)) or ventricular):ti,ab,kw 421444

#18 MeSH descriptor: [Noncommunicable Diseases] this term only 82

#19 ((non-communicable or non-infectious or noncommunicable or noninfectious or chronic) NEXT disease*):ti,ab,kw 26318

#20 MeSH descriptor: [Lung Diseases] explode all trees 57684

#21 (((lung* or pulmonary) NEAR/3 disease*) or cystic-fibrosis or asthma* or sarcoidos*):ti,ab,kw 69200

#22 MeSH descriptor: [Neoplasms] explode all trees 110900

#23 (cancer* or neoplasm*):ti,ab,kw 220658

#24 MeSH descriptor: [Diabetes Mellitus] explode all trees 45956

#25 (diabetes or diabetic or hyperglycemia* or glucose-intolerance):ti,ab,kw 116808

#26 MeSH descriptor: [Mental Disorders] explode all trees 98049

#27 (eating-disorder* or anorexia-nervosa or bulimi* or binge-eat* or (self NEXT (injur* or mutilat*)) or suicide* or suicidal or parasuicid* or mood-disorder* or affective-disorder* or bipolar or mania or manic or depression or depressive or dysthymi* or neurotic or neurosis or adjustment-disorder* or anxiety-disorder* or obsess* or compulsi* or panic or phobi* or ptsd or posttrauma* or post-trauma* or chronic-fatigue* or affective-symptoms or mental-disorder* or mental-health or schizo* or (mental* NEAR/2 ill*)):ti,ab,kw 170087

#28 #16 or #17 or #18 or #19 or #20 or #21 or #22 or #23 or #24 or #25 or #26 or #27 953103

#29 #15 and #28 with Publication Year from 2022 to 2023, in Trials Embase:2243, PubMed:1740, Cinahl:30

Search updated: 2024-06-05

Database: Ovid MEDLINE(R) and Epub Ahead of Print, In-Process, In-Data-Review & Other Non-Indexed Citations, Daily and Versions <1946 to June 05, 2024>

Search Strategy: 1 (tele* medicine* or telemedicine or tele* nurs* or telenurs* or tele* support* or telesupport* or tele* mental* or telemental* or telecare* or tele* care* or teleconsult* or tele* consult* or telefollow* or tele* follow* or telehealth* or tele* health* or telehome* or tele* home* or telemanag* or tele*manage* or telepatien* or tele* patient* or telesupport* or tele* support* or e-care* or ecare* or e-health* or ehealth* or e-medicine* or emedicine* or e-nursing* or enursing* or mnurs* or e-consult* or econsult* or e-mental* or emental* or mcare or mconsult* or m consult* or mhealth or mmedicine or mmental* or video conference* or videoconference* or video consult* or videoconsult* or iphone* or ipad* or smart device* or e-mail* or email* or ((digital* or tele* or remote* or distant*) adj3 (care* or healthcare* or consult* or observ* or check or checking or checkup* or treatment* or therap*)) or ((digital* or remote or distant or distance*) adj2 patient*) or ((text or phone) adj messaging) or ((mobile or smartphone or tablet) adj3 (app or apps or application*))).ti,ab,kf. (143664) 2 (home adj2 (monitoring* or technolog*)).ti,ab,kf. (5097) 3 Telemedicine/ or Telerehabilitation/ or Remote Consultation/ or Telenursing/ or Videoconferencing/ or

Telecommunications/ or Electronic Mail/ or Telephone/ or Cell Phone/ or Smartphone/ or Text Messaging/ or Mobile Applications/ (92625) 4 or/1-3 (192272) 5 exp Cardiovascular Diseases/ (2792694) 6 (cardio* or cardia* or heart* or coronary* or angina* or ventric* or myocard* or pericard* or isch?m* or emboli* or arrhythmi* or thrombo* or atrial fibrillat* or tachycardi* or endocardi* or sick sinus or hypertension* or stroke or ((vascular or cerebrovascular or arterial) adj (disease* or disorder*)) or ventricular).ti,ab,kf. (3649634) 7 Noncommunicable Diseases/ (3581) 8 ((non-communicable or non-infectious or noncommunicable or noninfectious or chronic) adj disease*).ti,ab,kf. (112185) 9 exp Lung Diseases/ (1283049) 10 (((lung* or pulmonary) adj3 disease*) or cystic fibrosis or asthma* or sarcoidos*).ti,ab,kf. (422901) 11 exp Neoplasms/ (3979051) 12 (cancer* or neoplasm*).ti,ab,kf. (2624296) 13 exp Diabetes Mellitus/ (527904) 14 (diabetes or diabetic or hyperglycemia* or glucose intolerance).ti,ab,kf. [sjekk staving] (835595) 15 exp Mental Disorders/ (1492875) 16 (eating disorder* or anorexia nervosa or bulimi* or binge eat* or (self adj (injur* or mutilat*)) or suicide* or suicidal or parasuicid* or mood disorder* or affective disorder* or bipolar or mania or

manic or depression or depressive or dysthymi* or neurotic or neurosis or adjustment disorder* or anxiety disorder* or obsess* or compulsi* or panic or phobi* or ptsd or posttrauma* or post trauma* or chronic fatigue* or affective symptoms or mental disorder* or mental health or schizo* or (mental* adj2 ill*)).ti,ab,kf. (1166287) 17 or/5-16 (12075777) 18 4 and 17 (84038) 19 randomized controlled trial.pt. (614486) 20 controlled clinical trial.pt. (95542) 21 (randomized or randomised).ti,ab. (838876) 22 randomly.ab. (434858) 23 (randomis* or randomiz* or randomly).ti,ab. (1195483) 24 groups.ab. (2687436) 25 (trial or study).ti. (2132198) 26 or/19-25 (5116141) 27 exp Animals/ (27241388) 28 Humans/ (22013161) 29 27 not (27 and 28) (5228227) 30 review.pt. (3333527) 31 meta analysis.pt. (201828) 32 news.pt. (225573) 33 comment.pt. (1036502) 34 editorial.pt. (693734) 35 cochrane database of systematic reviews.jn. (16670) 36 comment on.cm. (1036146) 37 (systematic review or literature review).ti. (313102) 38 or/29-37 (10241263) 39 26 not 38 [tilpasset cochrane sensitive rct] (4097613) 40 18 and 39 (27522) 41 limit 18 to "therapy (maximizes sensitivity)" (25582) 42 limit 41 to yr="2024 -Current" (1161) 43 limit 40 to yr="2024 -Current" (1739) 44 42 or 43 (2051)

Database: Embase <1974 to 2024 June 05>

Search Strategy: 1 (tele* medicine* or telemedicine or tele* nurs* or telenurs* or tele* support* or telesupport* or tele* mental* or telemental* or telecare* or tele* care* or teleconsult* or tele* consult* or telefollow* or tele* follow* or telehealth* or tele* health* or telehome* or tele* home* or telemanag* or tele*manage* or telepatien* or tele* patient* or telesupport* or tele* support* or e-care* or ecare* or e-health* or ehealth* or e-medicine* or emedicine* or e-nursing* or enursing* or mnurs* or e-

consult* or econsult* or e-mental* or emental* or mcare or mconsult* or m consult* or mhealth or mmedicine or mmental* or video conference* or videoconference* or video consult* or videoconsult* or iphone* or ipad* or smart device* or e-mail* or email* or ((digital* or tele* or remote* or distant*) adj3 (care* or healthcare* or consult* or observ* or check or checking or checkup* or treatment* or therap*)) or ((digital* or remote or distant or distance*) adj2 patient*) or ((text or phone) adj messaging) or ((mobile or smartphone or tablet) adj3 (app or apps or application*))).ti,ab,kf. (207081) 2 (home adj2 (monitoring* or technolog*)).ti,ab,kf. (7670) 3 *telemedicine/ or *telerehabilitation/ or *teleconsultation/ or *telenursing/ or *videoconferencing/ or *telecommunication/ or *e-mail/ or *telephone/ or *mobile phone/ or *smartphone/ or *text messaging/ or *mobile application/ (81228) 4 or/1-3 (248860)

5 exp *cardiovascular disease/ (3056636) 6 (cardio* or cardia* or heart* or coronary* or angina* or ventric* or myocard* or pericard* or isch?m* or emboli* or arrhythmi* or thrombo* or atrial fibrillat* or tachycardi* or endocardi* or sick sinus or hypertension* or stroke or ((vascular or cerebrovascular or arterial) adj (disease* or disorder*)) or ventricular).ti,ab,kf. (5042718) 7 *non communicable disease/ or exp *chronic disease/ (61504) 8 ((non-communicable or non-infectious or noncommunicable or noninfectious or chronic) adj disease*).ti,ab,kf. (151719) 9 exp *lung disease/ (1069793) 10 (((lung* or pulmonary) adj3 disease*) or cystic fibrosis or asthma* or sarcoidos*).ti,ab,kf. (615968) 11 exp *malignant neoplasm/ (3218127) 12 (cancer* or neoplasm*).ti,ab,kf. (3610713) 13 exp *diabetes mellitus/ (609019) 14 (diabetes or diabetic or hyperglycemia* or glucose intolerance).ti,ab,kf. [sjekk staving] (1265140) 15 exp *mental disease/ (1683266) 16 (eating disorder* or anorexia nervosa or bulimi* or binge eat* or (self adj (injur* or mutilat*)) or suicide* or suicidal or parasuicid* or mood disorder* or affective disorder* or bipolar or mania or manic or depression or depressive or dysthymi* or neurotic or neurosis or adjustment disorder* or anxiety disorder* or obsess* or compulsi* or panic or phobi* or ptsd or posttrauma* or post trauma* or chronic fatigue* or affective symptoms or mental disorder* or mental health or schizo* or (mental* adj2 ill*)).ti,ab,kf. (1506596) 17 or/5-16 (13989005) 18 4 and 17 (102918) 19 exp randomized controlled trial/ (827310) 20 (randomized or randomised).ti,ab. (1204581) 21 randomly.ab. (578860) 22 groups.ab. (3766496) 23 (trial or study).ti. (2628193) 24 or/19-23 (6682539) 25 18 and 24 (36028) 26 limit 25 to yr="2024 -Current" (1824) 27 limit 26 to embase (1018)

Database: Cochrane Register of Controlled Trials Search date: 2024-06-06

#1 ((tele* NEXT medicine*) or telemedicine or (tele* NEXT nurs*) or telenurs* or (tele* NEXT support*) or telesupport* or (tele* NEXT mental*) or telemental* or telecare* or (tele* NEXT care*) or (tele NEXT consult*) or teleconsult* or telefollow* or (tele* NEXT follow*) or telehealth* or (tele* NEXT health*) or telehome* or (tele* NEXT home*) or telemanag* or (tele NEXT manage*) or telepatien* or (tele* NEXT patient*) or

telesupport* or (tele* NEXT support*) or e-care* or ecare* or e-health* or ehealth* or e-medicine* or emedicine* or e-nursing* or enursing* or mnurs* or e-consult* or econsult* or e-mental* or emental* or mcare or mconsult* or m-consult* or mhealth or mmedicine or mmental* or video-conference* or videoconference* or video-consult* or videoconsult* or iphone* or ipad* or smart-device* or e-mail* or email* or ((digital* or tele* or remote* or distant*) NEAR/3 (care* or healthcare* or consult* or observ* or check or checking or checkup* or treatment* or therap*)) or ((digital* or remote or distant or distance*) NEAR/2 patient*) or ((text or phone) NEXT messaging) or ((mobile or smartphone or tablet) NEAR/2 (app or apps or application*))):ti,ab,kw 42184

#2 (home NEAR/2 (monitoring* or technolog*)) 1486

#3 MeSH descriptor: [Telemedicine] this term only 4057

#4 MeSH descriptor: [Telerehabilitation] this term only 340

#5 MeSH descriptor: [Remote Consultation] this term only 453

#6 MeSH descriptor: [Telenursing] this term only 46

#7 MeSH descriptor: [Videoconferencing] this term only 297

#8 MeSH descriptor: [Telecommunications] this term only 115

#9 MeSH descriptor: [Electronic Mail] this term only 440

#10 MeSH descriptor: [Telephone] this term only 2861

#11 MeSH descriptor: [Cell Phone] this term only 1050

#12 MeSH descriptor: [Smartphone] this term only 1166

#13 MeSH descriptor: [Text Messaging] this term only 1636

#14 MeSH descriptor: [Mobile Applications] this term only 1988

#15 #1 OR #2 OR #3 OR #4 OR #5 OR #6 OR #7 OR #8 OR #9 OR #10 OR #11 OR #12 OR #13 OR #14 45644

#16 MeSH descriptor: [Cardiovascular Diseases] explode all trees 155891

#17 (cardio* or cardia* or heart* or coronary* or angina* or ventric* or myocard* or pericard* or ischem* or ischaem* or emboli* or arrhythmi* or thrombo* or (atrial NEXT fibrillat*) or tachycardi* or endocardi* or sick-sinus or hypertension* or stroke or ((vascular or cerebrovascular or arterial) NEXT (disease* or disorder*)) or ventricular):ti,ab,kw 450963

#18 MeSH descriptor: [Noncommunicable Diseases] this term only 113

#19 ((non-communicable or non-infectious or noncommunicable or noninfectious or chronic) NEXT disease*):ti,ab,kw 28846

#20 MeSH descriptor: [Lung Diseases] explode all trees 64359

#21 (((lung* or pulmonary) NEAR/3 disease*) or cystic-fibrosis or asthma* or sarcoidos*):ti,ab,kw 72424

#22 MeSH descriptor: [Neoplasms] explode all trees 125042

#23 (cancer* or neoplasm*):ti,ab,kw 237406

#24 MeSH descriptor: [Diabetes Mellitus] explode all trees 46033

#25 (diabetes or diabetic or hyperglycemia* or glucose-intolerance):ti,ab,kw 125959

#26 MeSH descriptor: [Mental Disorders] explode all trees 106974

#27 (eating-disorder* or anorexia-nervosa or bulimi* or binge-eat* or (self NEXT (injur* or mutilat*)) or suicide* or suicidal or parasuicid* or mood-disorder* or affective-disorder* or bipolar or mania or manic or depression or depressive or dysthymi* or neurotic or neurosis or adjustment-disorder* or anxiety-disorder* or obsess* or compulsi* or panic or phobi* or ptsd or posttrauma* or post-trauma* or chronic-fatigue* or affective-symptoms or mental-disorder* or mental-health or schizo* or (mental* NEAR/2 ill*)):ti,ab,kw 186137

#28 #16 or #17 or #18 or #19 or #20 or #21 or #22 or #23 or #24 or #25 or #26 or #27 1025150

#29 #15 and #28 with Publication Year from 2023 to 2024, in Trials Embase: 1926, PubMed: 1815, Cinahl: 33

Appendix 2: Follow-up times in the included studies Follow-up times	Studies
1 month	Indraratna/Laursen/
1.5 months	Absolom/Hernandez-Quiles/Temple-Oberle/
2 months	Lee/Piotrowicz/
3 months	Absolom/Agarwal/Benzo/Dorsch/Faurholt-Jepsen/Garanin/Greer/ Hernandez-Quiles/Shimoyama/Widmer/Yannicelli

Appendix 3: GRADE assessments

Remote patient monitoring compared to usual care for health problem or population

Patient or population: health problem or population

Setting:

Intervention: Remote patient monitoring

Comparison: usual care

Outcomes	№ of participants (studies) Follow-up	Certainty of the evidence (GRADE)	Relative effect (95% CI)	Anticipated absolute effects	
				Risk with usual care	Risk difference with Remote patient monitoring
Proportion with hospitalizations	9068 (30 RCTs)	⊕⊕◯◯ Low[a,b]	**RR 0.86** (0.77 to 0.95)	322 per 1 000	**45 fewer per 1 000** (74 fewer to 16 fewer)
Number of hospitalizations	2391 (9 RCTs)	⊕⊕◯◯ Low[b]	-	The mean number of hospitalizations was **0.42**	MD **0.13 lower** (0.29 lower to 0.03 higher)
Hospital length of stay	4103 (12 RCTs)	⊕⊕◯◯ Low[c,d,e]	-	The mean hospital length of stay was **5.28** days	MD **0.84 days lower** (1.61 lower to 0.06 lower)
Mean number of outpatient visits	1989 (8 RCTs)	⊕⊕◯◯ Low[b,f]	-	The mean mean number of outpatient visits was **8.13**	MD **0.41 higher** (0.22 lower to 1.03 higher)

Remote patient monitoring compared to usual care for health problem or population					

Patient or population: health problem or population

Setting:

Intervention: Remote patient monitoring

Comparison: usual care

Outcomes	№ of participants (studies) Follow-up	Certainty of the evidence (GRADE)	Relative effect (95% CI)	Anticipated absolute effects	
				Risk with usual care	Risk difference with Remote patient monitoring
Proportion with outpatient visits	680 (6 RCTs)	⊕⊕⊕◯ Moderate[g]	**RR 0.94** (0.87 to 1.02)	585 per 1 000	**35 fewer per 1 000** (76 fewer to 12 more)
Number of emergency department visits	2464 (7 RCTs)	⊕◯◯◯ Very low[b,h]	-	The mean number of emergency department visits was **0.86**	MD **0** (0.31 lower to 0.32 higher)
Proportion with emergency department visits	2993 (11 RCTs)	⊕⊕◯◯ Low[i]	**RR 0.91** (0.79 to 1.05)	291 per 1 000	**26 fewer per 1 000** (61 fewer to 15 more)

*__The risk in the intervention group__ (and its 95% confidence interval) is based on the assumed risk in the comparison group and the **relative effect** of the intervention (and its 95% CI).

CI: confidence interval; **MD:** mean difference; **RR:** risk ratio

Remote patient monitoring compared to usual care for health problem or population

Patient or population: health problem or population

Setting:

Intervention: Remote patient monitoring

Comparison: usual care

Outcomes	№ of participants (studies) Follow-up	Certainty of the evidence (GRADE)	Relative effect (95% CI)	Anticipated absolute effects	
				Risk with usual care	Risk difference with Remote patient monitoring

GRADE Working Group grades of evidence
High certainty: we are very confident that the true effect lies close to that of the estimate of the effect.
Moderate certainty: we are moderately confident in the effect estimate: the true effect is likely to be close to the estimate of the effect, but there is a possibility that it is substantially different.
Low certainty: our confidence in the effect estimate is limited: the true effect may be substantially different from the estimate of the effect.
Very low certainty: we have very little confidence in the effect estimate: the true effect is likely to be substantially different from the estimate of effect.

Explanations

a. Only six of 30 studies have low risk of bias.

b. Significant Chi-Square. Effects in different directions.

c. Only 3 of 10 studies with low RoB.

d. CI from 2 less to 1 more.

e. Significant Egger's test.

f. Only 3 of 8 with low RoB.

g. Only 1 of 6 with low RoB

h. Almost all studies have high risk of bias or some concerns.

i. No study with low RoB.
